# Synthetic Rhamnolipid Bolaforms trigger an innate immune response in *Arabidopsis thaliana*

**DOI:** 10.1038/s41598-018-26838-y

**Published:** 2018-06-04

**Authors:** W. Patricio Luzuriaga-Loaiza, Romain Schellenberger, Yannick De Gaetano, Firmin Obounou Akong, Sandra Villaume, Jérôme Crouzet, Arnaud Haudrechy, Fabienne Baillieul, Christophe Clément, Laurence Lins, Florent Allais, Marc Ongena, Sandrine Bouquillon, Magali Deleu, Stephan Dorey

**Affiliations:** 1 RIBP-EA 4707, SFR Condorcet FR CNRS 3417, University of Reims Champagne-Ardenne, Reims, 51100 France; 2 ICMR, UMR CNRS 7312, SFR Condorcet FR CNRS 3417, University of Reims Champagne-Ardenne, Reims, 51100 France; 3 LBMI laboratory, Gembloux Agro-Bio Tech, SFR Condorcet FR CNRS 3417, University of Liège, Gembloux, B-5030 Belgium; 4 MiPI laboratory, Gembloux Agro-Bio Tech, SFR Condorcet FR CNRS 3417, University of Liège, Gembloux, B-5030 Belgium; 5 Chaire Agro-Biotechnologies Industrielles (ABI), AgroParisTech, CEBB, Pomacle, 51110 France

## Abstract

Stimulation of plant innate immunity by natural and synthetic elicitors is a promising alternative to conventional pesticides for a more sustainable agriculture. Sugar-based bolaamphiphiles are known for their biocompatibility, biodegradability and low toxicity. In this work, we show that Synthetic Rhamnolipid Bolaforms (SRBs) that have been synthesized by green chemistry trigger *Arabidopsis* innate immunity. Using structure-function analysis, we demonstrate that SRBs, depending on the acyl chain length, differentially activate early and late immunity-related plant defense responses and provide local increase in resistance to plant pathogenic bacteria. Our biophysical data suggest that SRBs can interact with plant biomimetic plasma membrane and open the possibility of a lipid driven process for plant-triggered immunity by SRBs.

## Introduction

Plant innate immunity is mediated by the perception of invasion patterns (IPs), originating from the pathogens or the plant, by plant IP receptors (IPRs)^1^. Early signalling events of the IP-triggered response (IPTR) include the release of reactive oxygen species (ROS), intra/extracellular Ca^2+^ and K^+^ ion fluxes, medium alkalinisation of the apoplast and activation of cytoplasmic protein kinases including mitogen-activated protein kinases (MAPKs) and Ca^2+^-dependent protein kinases (CDPKs)^2^. Crosstalk of plant hormones including salicylic acid (SA), jasmonic acid (JA), ethylene (ET), abscisic acid (ABA) and brassinosteroids (BR) differentially regulates transcriptional reprogramming leading to plant defence gene activation^3,4^. Ultimately, plant immune response results in the strengthening of cell walls, the production of antimicrobial compounds and in some cases an hypersensitive reaction (HR) that altogether restrict pathogen growth^5^.

Exogenous IPs from pathogenic origin known as Microbe-Associated Molecular Patterns (MAMPs) are represented by a wide variety of structurally distinct molecules including flagellin peptides, peptidoglycans, lipopolysaccharides from bacteria or chitin and β-glucans from fungi and oomycetes^5,6^. The natural bacterial amphiphilic compounds rhamnolipids and lipopeptides have also been characterized as a new class of MAMPs^7–9^.

Synthetic elicitors are small compounds, structurally distinct from IPs that can trigger plant immune responses by mimicking IPs perception or IPs-triggered plant signalling. In addition, they can induce plant protection against pathogens without being directly toxic to the microorganism^10^. Several classes of synthetic elicitors have been characterized so far including low molecular weight polyacrylic acid derivatives, imprimatins, sulfonamides, adipic acid derivatives or SA and JA analogs^10^. 2,6-dichloro-isonicotinic acid (INA) and benzo (1,2,3) thiadiazole-7-carbothioic acid *S*-methyl ester (BTH) (also known as Bion®) are the best-known SA analogs that mimic SA-triggered immune responses without its deleterious effects^11–14^. Recently, 2-(5-bromo-2-hydroxy-phenyl)-thiazolidine-4-carboxylic acid (BHTC) that induces plant disease resistance against bacterial, oomycete, and fungal pathogens was shown to link plant immunity to hormesis^15^. Another recently discovered synthetic elicitor, the 3,5-dichloroanthranilic acid (DCA) induces NPR1-dependent and NPR1-independent mechanisms of disease resistance against the pathogenic oomycete *Hyaloperonospora arabidopsidis* (*Hpa*) and the bacterial pathogen *Pseudomonas syringae* pv. *tomato* DC3000 (*Pst*) in *Arabidopsis thaliana* (hereafter, *Arabidopsis*)^16^. DPMP (2,4-dichloro-6-{(*E*)-[(3-methoxyphenyl)imino]methyl} phenol) triggers a robust immune response in *Arabidopsis* and tomato^17^. Synthetic amphiphilic molecules are also able to stimulate plant defence responses, as exemplified by short cationic lipopeptides^18^ or lipid diC_14_^19^.

In the last few years, attention to amphiphilic molecules has increased because of their multiple applications in different areas including bioremediation, pharmacology, medical devices sanitization and agriculture^20–24^. Recent researches have been focused on D-xyloside-based and L-rhamnoside-based bolaamphiphiles surfactants characterized by their biocompatibility, biodegradability or low toxicity and targeted for the development of efficient and low cost lipid-based drug delivery systems^25–29^. The configuration of the bolaamphiphile surfactants consists of a long hydrophobic spacer connecting two hydrophilic entities; the molecules are more water soluble than the average surfactant and their properties make them extremely suitable for applications in nanotechnology, electronics, and gene and drug delivery^30^. Synthetic xylolipid bolaforms (SXBs) and rhamnolipid bolaforms (SRBs) containing a C_18_ acyl chain have recently shown to interact with mammalian-based biomimetic systems of plasma membranes^31^.

In the present work, we investigated the potential of SRBs as new synthetic elicitors in plants. We show that SRBs are perceived by *Arabidopsis* and trigger an atypical plant immune response. We also show that the increase in resistance to the hemibiotrophic pathogen *Pst* depends on the fatty acid chain length of SRBs. Moreover, our results suggest that direct interaction of SRBs with the lipid fraction of plasma membrane could participate in their perception by plants.

## Results

### Synthesis of SRBs

**Figure 1:**
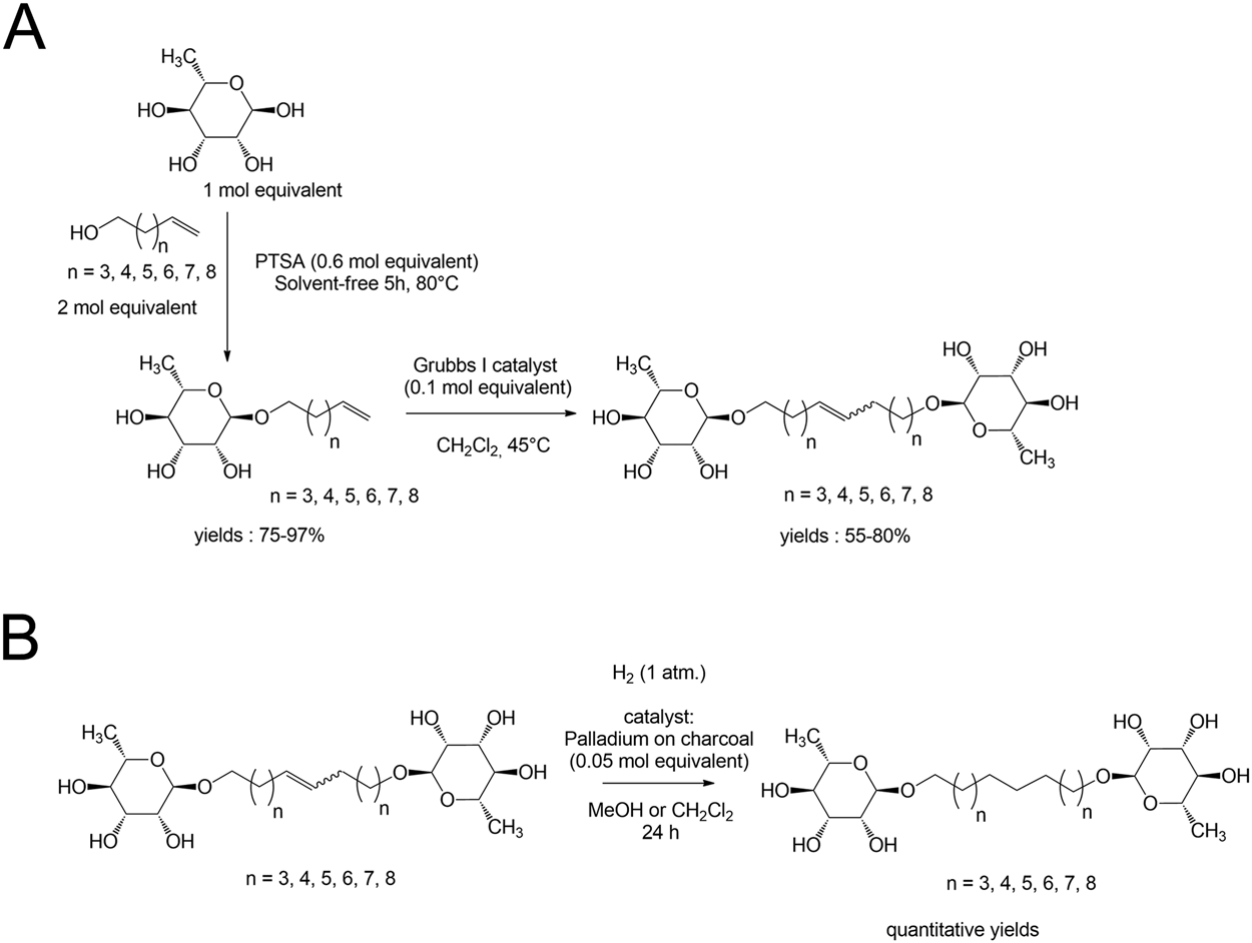
(**A**) Synthesis of unsaturated synthetic rhamnolipid bolaforms (SRB_Xi_). (**B**) Synthesis of saturated synthetic rhamnolipid bolaforms (SRB_X_).

The efficient synthesis of symmetric bolaamphiphiles derived from L-rhamnose SRBs (Fig. 1A) has been realized using green chemistry principle (principle 5 i.e. “Safer Solvents and Auxiliaries”, principle 7 i.e. “Use of Renewable Feedstocks” and principle 9 i.e. “Catalysis”)^29,32^. Glycosidations were performed without solvent because the alcohols can play this role; excess of alcohol could be then removed and recycled for another reaction batch. Moreover, this method led to rhamnosides with a shorter reaction time and high yields (75–85%). Finally, the metathesis steps were performed in the presence of Grubbs I catalyst, without protecting steps of the OH functions, in methylene chloride alone without addition of methanol. The unsaturated SRBs could be then hydrogenated through classical Pd-catalyzed reactions and led to saturated SRBs surfactants (Fig. 1B). As lipid elicitors including lipopeptides and plant or microorganism-derived fatty acids have been shown to induce immune responses that depend on the chain length, unsaturation degree and position of the double bond in the fatty acid chain^8,33–36^, we selected six SRBs with a fatty acid chain length of C_10_, C_14_ or C_18_, saturated (SRB_10_, SRB_14_, SRB_18_, respectively) or unsaturated (SRB_10i_, SRB_14i_, SRB_18i_, respectively) for the following experiments.

### SRBs are perceived by *Arabidopsis* and display unconventional signalling-related immune responses

**Figure 2:**
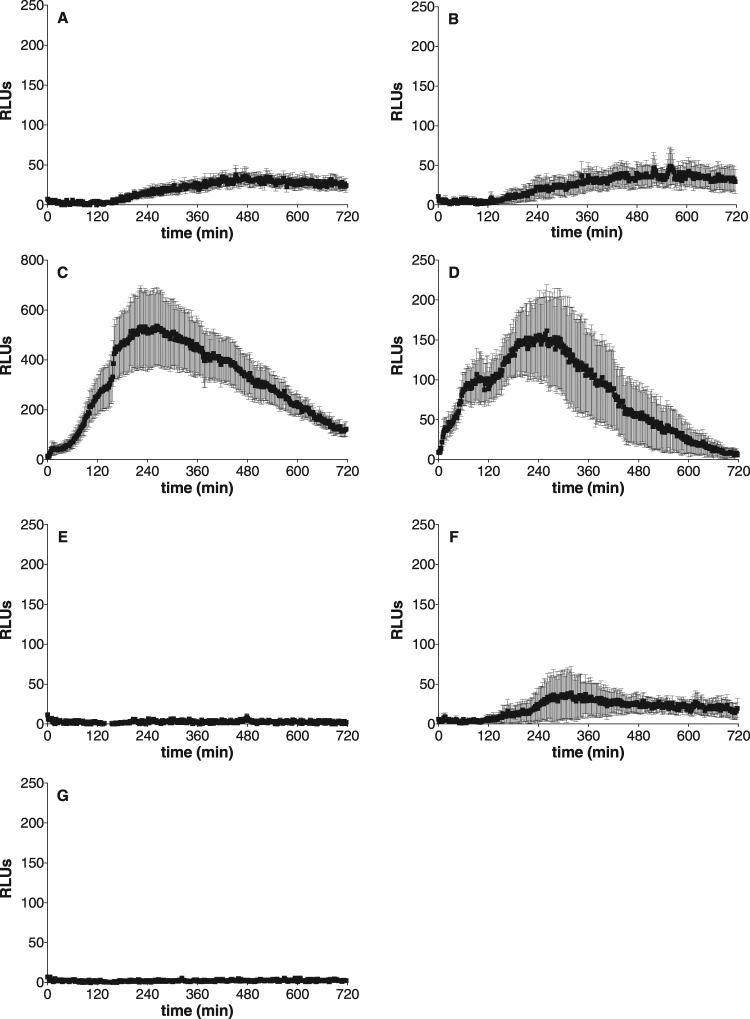
Extracellular ROS production upon elicitation of *Arabidopsis* with unsaturated SRBs. Petioles of 6-weeks-old wild type *Arabidopsis* plants were placed in a 96-wells plate and incubated in water overnight prior SRB elicitation. For ROS monitoring, a luminol-peroxidase solution containing 100 µM, or 350 µM of the corresponding SRB or solvent (0.5% ethanol) was added to each well. All SRBs contain the same amount of ethanol. The luminescence was read immediately after elicitation every 2 min with a Tecan Infinity F200 PRO for 720 min. Data presented are means of at least triplicate experiments ± standard error of the mean (SEM) with n = 6 for each experiment. (**A**,**C,E**) SRB_10i_, SRB_14i_ and SRB_18i_ at 100 µM, respectively. (**B**,**D**,**F**) SRB_10i_, SRB_14i_ and SRB_18i_ at 350 µM, respectively. (**G**) Control (ethanol).

**Figure 3:**
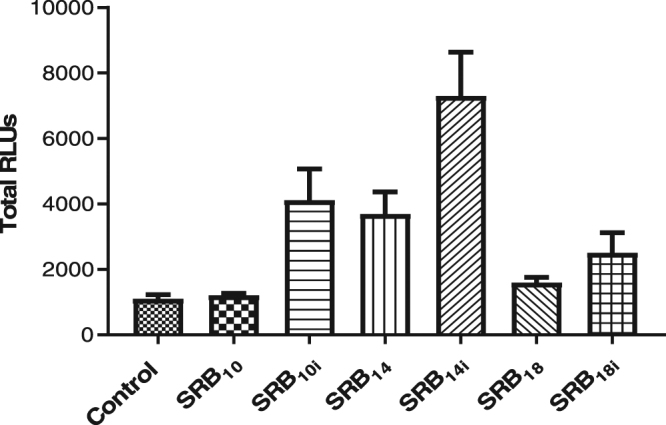
Comparison of extracellular ROS production upon elicitation of *Arabidopsis* with saturated and unsaturated SRBs. Petioles of 6-weeks-old wild type *Arabidopsis* plants were placed in a 96-wells plate and incubated in water overnight prior SRB elicitation. For ROS monitoring, a luminol-peroxidase solution containing 350 µM of the corresponding SRB or solvent (0.5% ethanol) was added to each well. All SRBs contain the same amount of ethanol. The luminescence was read immediately after elicitation every 2 min with a Tecan Infinity F200 PRO for 720 min. Histograms were calculated as the total RLUs over 12 hours of monitoring. Data presented are means of at least triplicate experiments and SEM with n = 6 for each experiment.

In order to investigate whether SRBs are perceived by plants and could induce an immune response, leaves or petioles from *Arabidopsis* were challenged with the different SRBs and monitored for production of extracellular ROS, a widely used marker of plant immunity^37^. Although ROS production gave similar profiles on both organs, the test was more robust and sensitive with petioles that were therefore used in the following ROS experiments (Supplementary Fig. S1). All unsaturated SRBs induced a sustained ROS production when applied at 350 µM, the minimal concentration necessary to induce local plant protection using natural rhamnolipids^9^, suggesting that all these molecules could be perceived by *Arabidopsis* (Fig. 2B,D,F). For all molecules, the response lasted for several hours and tended to return to basal levels 12 hours after treatment. By contrast, the canonical elicitor flg22 induced a rapid and transient ROS production immediately after plant treatment in our experimental conditions (Supplementary Fig. S2)^38^. SRB_10i_ and SRB_14i_ also stimulated a long lasting ROS burst at 100 µM in contrast to SRB_18i_ that was inactive at this concentration (Fig. 2A,C,E). Moreover, SRB_14i_ induced the earliest and highest response when compared to other SRBs (Fig. 2; Supplementary Fig. S3). Different levels of response between saturated/unsaturated SRBs were observed. SRB_14i_ were more active than SRB_14_ and only SRB_10i_ and SRB_18i_ displayed a significant response on ROS assays (Fig. 3).

**Figure 4:**
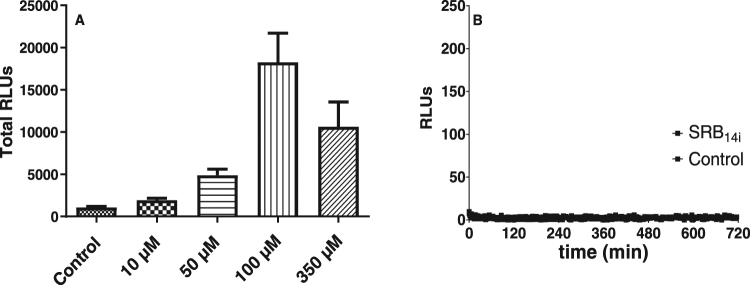
Dose response and rbohD dependence of extracellular ROS production induced by SRB_14i_. (**A**) Petioles from wild type *Arabidopsis* were challenged with different concentrations of SRB_14i_ or 0.5% ethanol (control) and monitored for extracellular ROS production for 720 min. Histograms were calculated as the total RLUs over 12 hours of monitoring. Data presented are means of at least triplicate experiments and SEM with n = 6 for each experiment. (**B**) Extracellular ROS production in *rbohD* mutant challenged with the SRB_14i_ at 350 µM or ethanol (control). Data presented are means of at least triplicate experiments ± standard error of the mean (SEM) with n = 6 for each experiment.

**Figure 5:**
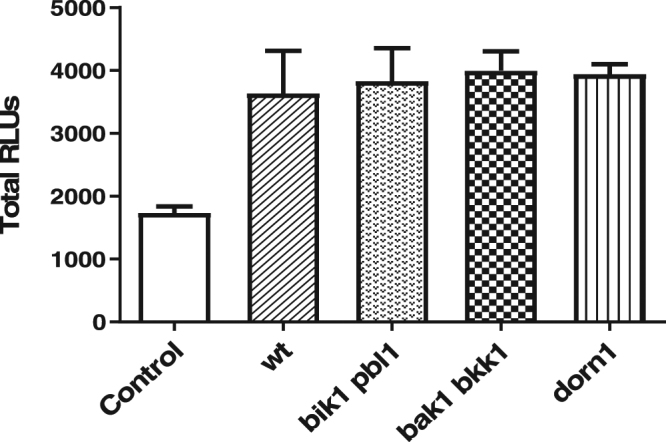
SRB_14i_-triggered extracellular ROS production in *Arabidopsis* RLK mutants. Histograms were calculated as the total RLUs over 12 hours of monitoring. Data represent the mean and SEM of two independent experiments with n = 6 for *wt, bik1-pbl1, bak1-5-bkk1-1* or *dorn1-1* mutants elicited with SRB_14i_ at 350 µM or 0.5% ethanol for the control.

Dose response experiments on SRB_14i_ showed that the minimal concentration necessary for this molecule to induce a robust ROS response in *Arabidopsis* was 50 µM (Fig. 4A). SRB_14i_-triggered ROS production was fully dependent on the membrane bound NADPH oxidase RBOHD^39^ as no ROS production could be detected in *rbohD* mutant plants (Fig. 4B). Receptor-like kinases (RLKs) are key components required for activation of the immune response following IP perception^40^. We monitored SRB_14i_-mediated ROS production in the mutants *bak1-5-bkk1-1*^41^ and *bik1-pbl1*^42^ which are essential nodes involved in IP-triggered immunity. In addition, we used *dorn1-1* mutant to investigate the sensing of extracellular ATP. ATP is among the molecules that are released by cell damage, and recent evidence suggests that ATP can serve as damage-associated molecular patterns (DAMPs)^43,44^. Compared to wild type (wt) plants, none of these mutants displayed a significant decrease in ROS production (Fig. 5) after SRB_14i_ perception, suggesting that signal transduction following perception of the synthetic elicitor does not involve these RLKs.

**Figure 6:**
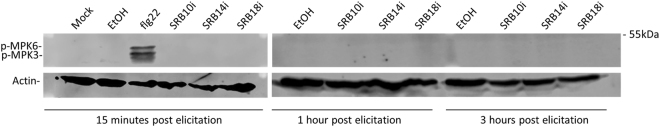
MAPK3 and 6 phosphorylation of SRBs-elicited *Arabidopsis*. Leaf discs of *Arabidopsis* were elicited with 1 µM flg22, 350 µM of unsaturated SRBs, 0.5% ethanol or water (mock) for 15 min, 1 h, or 3 h. Kinase activation is shown by immunoblot analysis using an anti-p44/42-ERK antibody. Individual MPKs are identified by molecular mass and indicated by arrows. Anti-actin antibodies were used for protein quantification for each sample. Experience has been done twice with similar results.

MAP kinases are also important signalling components involved in immune related signalling^45^. More specifically, MAPK3 and MAPK6 phosphorylation is a key process related to immunity signalling upon perception of canonical IP elicitors^2^. None of the MAP kinases were activated after SRB_10i_, SRB_14i_ or SRB_18i_ perception at 100 µM or 350 µM when compared to control 15, 60 or even 180 minutes post-treatment, the latest time point corresponding to the peak of ROS production (Fig. 6, Supplementary Fig. S4).

### SRBs differentially induce immunity-related gene markers and electrolyte leakage in *Arabidopsis*

**Figure 7:**
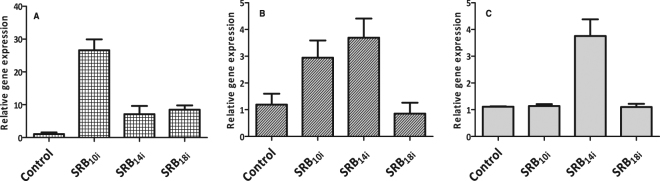
SRBs-triggered immune gene expression. A pool of five 10-days-old *Arabidopsis* seedlings were elicited with 100 µM SRBs or 0.5% ethanol (control) and collected for RNA extraction and RT-qPCR 9 hours post-elicitation. Transcript expression was normalized to control plants at 0 hour post-treatment. *CYP71A12* (**A**), *PDF1.2* (**B**) and *NPR1* (**C**) transcripts were analyzed. Results show the mean and SEM of two independent experiments.

To assess the capacity of SRBs to induce transcriptomic changes related to *Arabidopsis* immunity, we monitored the expression of *PDF1.2, NPR1* and *CYP71A12* genes. *PDF1.2* is a well-known plant defensin gene activated concomitantly by the JA and ET pathways^46,47^ while *NPR1* is an important regulator of PR proteins linked to the SA pathway^48,49^. *CYP71A12* encodes a cytochrome P450, which catalyzes the conversion of indole-3-acetaldoxime to indole-3-acetonitrile during biosynthesis of the phytoalexin camalexin^50^. RT-qPCR was performed 9 hours after elicitation on seedlings elicited with SRB_10i_, SRB_14i_ or SRB_18i_. All SRBs were able to significantly induce *CYP71A12* whereas only SRB_10i_ and SRB_14i_ stimulated *PDF1.2* expression (Fig. 7A,B). Interestingly, the SA dependent *NPR1* gene was only activated after SRB_14i_ challenge (Fig. 7C).

**Figure 8:**
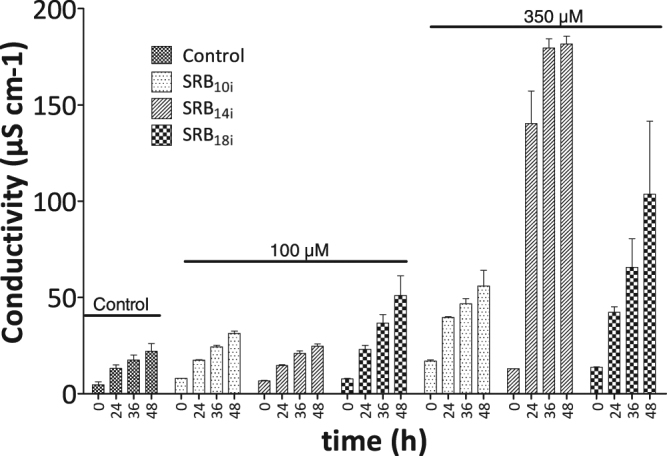
Electrolyte leakage induced by SRBs on *Arabidopsis* leaf discs. Leaf disks of 6-weeks-old *Arabidopsis* plants were incubated in water overnight prior SRB elicitation. Leaf discs were then challenged with 100 µM or 350 µM of SRBs or 0.5% ethanol. Electrolyte leakage was monitored with a conductivity meter. Data represent the mean and SEM of three independent experiments with n = 5 for each experiment.

We also performed conductivity measurements on *Arabidopsis* leaf discs to assess if SRBs induced changes in plant plasma membrane permeability (Fig. 8). At 350 µM, SRB_14i_ triggered a strong electrolyte leakage in *Arabidopsis* leaves within 24 hours post-treatment. SRB_10i_ and SRB_18i_ treatment also increased medium conductivity, SRB_18i_ being the most active, but at a lower extent than SRB_14i_ (Fig. 8). At 100 µM, only SRB_18i_ provoked a slight electrolyte leakage response. Despite the electrolyte leakage responses, we did not observe significant phytotoxicity effects (chlorosis or necrotic spots) on *Arabidopsis* plants infiltrated with SRBs even at the highest concentration (Supplementary Fig. S5).

### SRB_14i_ triggers local increase in resistance to *Pst* but not to *Botrytis cinerea*

**Figure 9:**
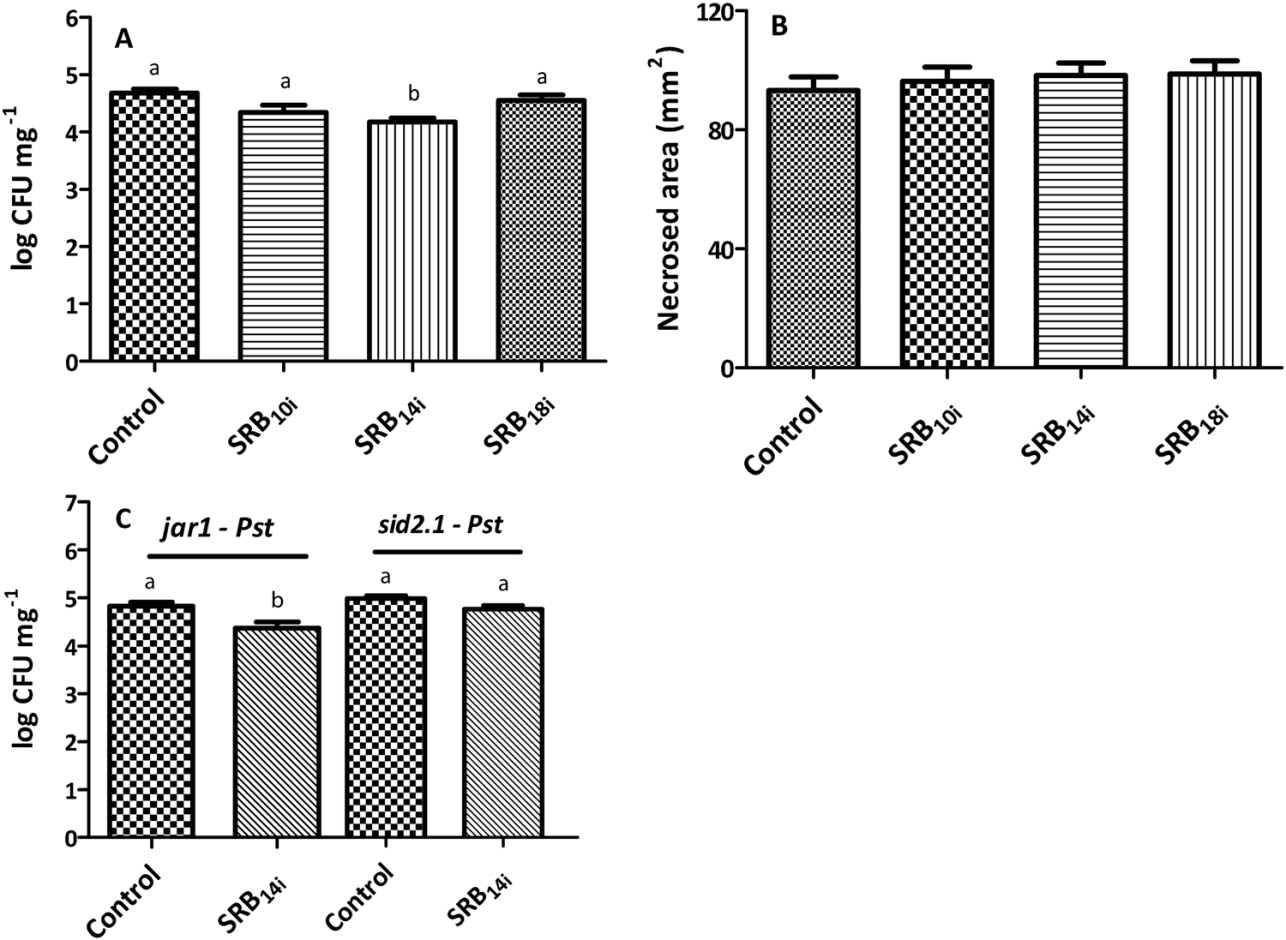
Plant protection induced by SRBs on wild type and hormonal *Arabidopsis* mutants. Plants were spray-elicited with 350 µM SRBs or 0.5% ethanol (control) 48 hours prior pathogen inoculation. SRB protection in wt *Arabidopsis* (**A**)*, jar1* and *sid2* mutants (**C**) against *Pst*. *Arabidopsis* clumps (15 plants per pot) were spray-inoculated and bacterial titers of infected tissues were determined three days after inoculation. Results represent the mean and SEM of three independent experiments with n ≥ 24 (**A**) and n ≥ 20 (**C**). The differences were analyzed by the non-parametric Kruskal-Wallis test (p < 0.05) for (**A**) and by the Mann Whitney test for (**C**). SRB protection in wt *Arabidopsis* against *Botrytis cinerea* (**B**). *Arabidopsis* leaf discs from six-weeks-old plants were drop-inoculated with 10 µL of *B. cinerea* conidia suspension (10^5^ conidia/mL) and incubated in Petri dishes containing water-wet filter paper. The area of necrosis was determined with ImageJ software four days after inoculation. Data represent the mean and SEM of three independent experiments with n ≥ 20 for each experiment. No significant differences were observed by the non-parametric Kruskal-Wallis test (p < 0.05).

In order to investigate whether SRBs are able to trigger local induced resistance to pathogens, we performed protection experiments using two different lifestyle microorganisms, the hemibiotrophic bacteria *Pst* and the necrotrophic fungus *B. cinerea*^51,52^. For the protection test against *Pst*, *Arabidopsis* plants were sprayed with SRBs and spray-inoculated with *Pst* two days later. Three days after inoculation leaf samples were collected for *Pst* CFU titration. SRB_14i_ but not SRB_10i_ or SRB_18i_, induced significant disease reduction against *Pst* (Fig. 9A). SRB_i_ did not show any bacteriostatic or bactericidal effect on *Pst* when applied at 350 µM (Supplementary Fig. S6), suggesting that the increased resistance was mediated by plant defense responses. To monitor *B. cinerea* infection, leaf discs pre-treated with SRBs were inoculated two days later with a conidia suspension (10^5^ conidia/mL) and four days after inoculation, the necrotized leaf area was measured. As shown in Fig. 9B, none of the SRBs induced plant protection against the necrotrophic fungus.

To investigate the hormone signalling pathways involved in SRB_14i_-driven increase in resistance to *Pst*, we performed protection experiments in *sid2*^53,54^ and *jar1*^55^ mutants plants impaired in SA and JA signalling, respectively^9^. Enhance protection to *Pst* observed in wt plants was lost in the *sid2* mutant, demonstrating that the SA signalling pathway is involved in SRB_14i_-triggered immunity to the pathogen. JA signalling pathway is however not involved in the process as the increase in resistance to *Pst* was conserved in *jar1* mutant (Fig. 9C).

### SRB_14i_ interacts with plant biomimetic plasma membrane

**Figure 10:**
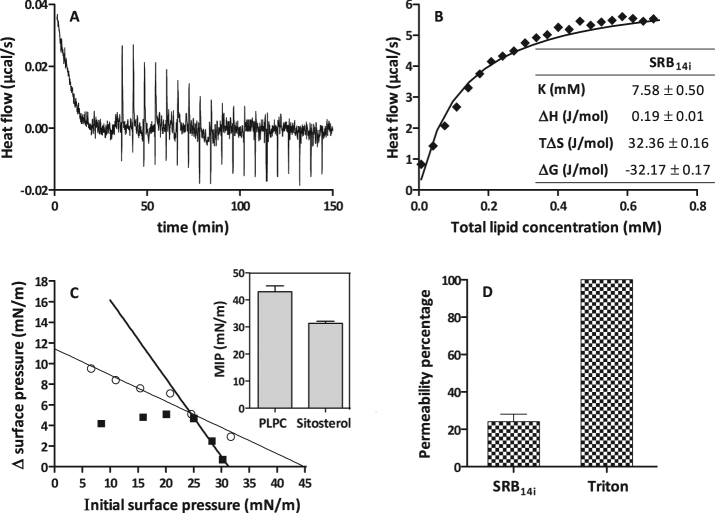
Interaction of SRB_14i_ with plant biomimetic plasma membranes. (**A**) Example of ITC raw data. Heat flow (µcal/s) versus time (min) profile resulting from injection of 10 µL aliquots of PLPC/Sitosterol vesicles (5 mM) into the reaction cell containing SRB_14i_ solution (100 µM) in Tris buffer (pH 7.4) at 26 °C. (**B**) Cumulative heats vs. total lipid concentration as obtained from SRB_14i_ (100 µM) titration with PLPC/Sitosterol vesicles at 26 °C. The solid lines correspond to theoretical fits of the total cumulative heat. B Inset) Thermodynamic parameters for the binding of SRB_14i_ to PLPC/sitosterol (80/20) vesicles at 26 °C. Data obtained from the fitting of the cumulative heats vs. total lipid concentration plot. K = binding coefficient, ΔH = molar enthalpy change corresponding to the transfer of SRB_14i_ from the aqueous phase to the bilayer membrane, ΔS = molar entropy change, ΔG = free energy. (**C** left) Adsorption of SRB_14i_ into a lipid monolayer. Surface pressure increases as a function of the initial surface pressure of the PLPC or sitosterol monolayers. SRB_14i_ is injected beneath the lipid monolayer at a final concentration of 4.5 μM in the Tris-subphase at pH 7.4 and 22 ± 1 °C. The solid line represents the linear fitting of the data (R^2^ = 0.96 and 0.99 for PLPC and Sitosterol, respectively). PLPC is represented by circles and sitosterol by squares. (**C** right) Maximal Insertion Pressure (MIP) of the SRB_14i_ into a PLPC or sitosterol monolayer. The MIP was obtained by linear regression of the plot Δ∏ = f (∏i) plot with the x-axis. (**D**) Determination of the permeabilization capacity of SRB_14i_. Vesicles composed of PLPC/Sitosterol (80/20) containing calcein were challenged with 10 µM SRB_14i_ and monitored for calcein fluorescence de-quenching for 15 min. Data represent the mean and SD of two independent experiments and were normalized to Triton (100% permeabilization) and DMSO (0%).

The atypical immune signature of SRBs raised the hypothesis of a direct interaction of the molecules with lipids from the plasma membrane instead of a receptor-dependent perception, as it has already been proposed for the amphiphilic bacterial elicitor surfactin^33^. In order to assess the affinity of SRB_14i_ for plasma membrane lipids, its partitioning into PLPC/sitosterol (80:20) vesicles and its insertion into PLPC or sitosterol monolayers were determined by ITC and Langmuir monolayer experiments. ITC raw data (Fig. 10A) displayed a gradual decrease of the positive heat flow signal over the course of the successive LUV injections. This profile is typical of a binding phenomenon. The thermodynamic parameters obtained from the fitting of the cumulative heat vs lipid concentration plot (Fig. 10B) indicated that the binding reaction of SRB_14i_ with vesicles is spontaneous (ΔG < 0), endothermic (ΔH > 0) and leads to a positive change of the entropy (ΔS > 0) (Fig. 10B). The absolute value of entropy change is much higher than the absolute values of enthalpy change indicating that the binding is notably driven by hydrophobic interactions^56^. ITC results showed that SRB_14__i_ could bind to liposomes and thus suggest an interaction of SRB_14i_ with the lipid phase of the plant plasma membrane. As observed in Fig. 10C, SRB_14i_ was preferably inserted into PLPC than into sitosterol monolayers (MIP_PLPC_ > MIP_sitosterol_) but both systems are compatible with the SRB_14i_ membrane insertion. In addition, SRB_14i_ also induced 20–30% vesicle permeabilization, when applied at 10 µM (Fig. 10D), suggesting a transient perturbation of the bilayer.

## Discussion

In this study, we show for the first time that the synthetic bolaamphiphilic glycolipids SRBs are perceived by *Arabidopsis* cells and induce an immune response characterized by unconventional signalling events, defence gene activation and enhanced resistance to the hemibiotrophic pathogen *Pst*. In the last years, the advancement in combinatorial and organic chemistry has boosted the interest on synthetic compounds with plant eliciting capacities since they have been demonstrated to be effective in control of plant pathogens and to induce plant immunity as efficiently as natural elicitors^10^. A recent publication reported a large screening identifying 114 synthetic elicitors that activate expression of the pathogen-responsive CaBP22−333::GUS reporter gene in *Arabidopsis*; 33 of which being [(phenylimino)methyl]phenol (PMP) derivatives or PMP-related compounds^17^. Their chemical preparation is relatively difficult and costly due to the starting materials and the synthetic procedures involving the use of environmental unfriendly compounds and solvents. As previously described, the synthesis of SRBs follows the principles of green chemistry and uses biosourced materials. Interestingly, most of the synthetic elicitors described so far are aromatic compounds exhibiting at least a benzene ring^10^. All these compounds induce disease resistance to pathogens. However, their mode of action can strongly differ depending on the structures of the molecules. For instance, DCA transiently induces defence reactions to *Hpa* and *Pst* and DCA-induced resistance to *Hpa* is partially dependent on the NPR1 pathway^16^. This is in contrast with SA analogues like INA or BTH that fully involve NPR1 to induce a broad range resistance to biotrophs^12,57,58^. BHTC induces *Arabidopsis* disease resistance against bacterial, oomycete, and fungal pathogens. BHTC-triggered protection to *Hpa* is independent from SA pathway but requires NPR1^15^. DPMP mode of action is distinct from that of DCA and similar to BHTC, since its ability to induce immunity against *Hpa* is completely blocked in *npr1* mutant plants^17^. Sulfonamides like sulfamethoxazole are potent inducers of plant immunity against *Pst* that do not require NPR1-dependent canonical SA defence pathway^59,60^. Apart from aromatic-derived elicitors, a synthetic cationic lipid diC_14_ with amphiphilic properties, and therefore more related to SRBs, has recently been described as a potent elicitor^19^. Like SRB_14i_, diC_14_ enhances plant protection to *Pst* but not to *B. cinerea* and induced resistance to *Pst* is SA-dependent but JA-independent. Moreover, the amidine headgroup and chain length were important for its activity.

Interestingly, all the synthetic elicitors characterized so far are acting at relatively high concentrations (generally from 100 µM to 1 mM) compared to canonical MAMP/IPs like flagellin, active at micromolar or even nanomolar ranges (supplementary Fig. S2)^5,10^. This is also the case for SRBs, inducing defence reactions like ROS production at 50–100 µM. This could be explained by a hormone-like mode of action or a different perception mechanism not involving plant receptors.

To our knowledge, little data exist on the signalling events involved in synthetic elicitor-triggered immunity. Here, we show that SRB_14i_ induces early and sustained ROS production. ROS production is a characteristic immune-related signalling event following IP perception^37^. In *Arabidopsis*, ROS production during the immune response is mainly achieved by the NADPH oxidase RBOHD^39^. However, a recent report on LPS perception also suggested that long lasting ROS production could originate from chloroplasts^61^. We found that sustained ROS production triggered by unsaturated SRBs was fully dependent on RBOHD demonstrating that a long lasting oxidative burst could also be generated by the membrane bound NADPH oxidase. These results also demonstrate that SRB-driven ROS accumulation is related to an active process and not a direct result of potential release after cell damages. MAPK3 and 6 are usually involved in MAMP-triggered immunity^2^. Interestingly, although ROS production was triggered by SRBs, we did not observed activation of these canonic MAPKs in our conditions.

Regarding the cell perception of SRBs, we have demonstrated that the chemical structure of SRBs, in particular the length and saturation of the acyl chain, is important for the plant perception and induction of plant defences. We observed that the most active SRB possessed a C_14_ acyl chain with an unsaturation (SRB_14i_). When the SRB_14i_ was assessed in ITC experiments, we observed that it effectively bounds to the model membrane *via* hydrophobic interactions. The importance of the saturation and chain length has been proposed for arachidonic acid and derivatives in potato tubers elicitation^34^. Acyl chain of 20 carbons resulted in effective elicitation by contrast to C_16_, C_18_ or C_22_, which were inactive or showed a very low eliciting activity. By contrast, synthetic ultrashort cationic lipopeptides were more active with chains containing 16 carbons^18^. Cambiagno *et al*. noticed that diC_14_ was more efficient in inducing plant resistance than diC_16_^19^. Lipopeptides such as orfamide or surfactin, both amphiphilic elicitors, display an immune response that depends on the concentration and/or the length of the fatty acid chain^8,33,62,63^. Surfactin with 14 or 15 carbons were more efficient than the C_12_ and C_13_ counterparts^8,33^. Orfamide plant eliciting activity was however not dependent on the chain length but rather on the concentrations of the molecules depending on the plant species^62–64^.

Plasma membrane perturbation and lipid signalling play an important role in the adaptation to biotic and abiotic challenges, especially by modifying the compartmentalization, distribution, abundance and type of lipids and proteins present in the membrane upon stress perception^65,66^. Our results on ITC experiments suggest that SRB_14i_ has the ability to insert into plasma membrane and this insertion could result in plasma membrane reorganization. This membrane perturbation attested by the biophysical permeabilization experiments could in turn result in electrolyte leakage and plant defense activation. Savchenko and colleagues^35^ demonstrated that even minor modifications of the plant cell lipid composition with exogenous fatty acids derived from animals or pathogens induced plant defence activation. Some MAMPs have also been reported to alter the plasma membrane state. Tobacco and *Arabidopsis* cells treated with flg22, oligogalacturonides or cryptogein induced an increase of lipid self-association in specific domains of the plasma membrane upon elicitation coinciding with the onset of plant defence signalling^67,68^. The same reports state that cryptogein enhances ROS production by recruiting plant sterols from the plasma membrane increasing its fluidity. Similarly, it has been observed that surfactin favourably interacts with phospholipids, suggesting that surfactin insertion into the plasma membrane participates in the elicitation mechanism^33^. Synthetic rhamnolipids with modified carboxylated fatty acid chains were recently shown to differentially induce ROS production^69^. Alkylated rhamnolipids induced a higher response than carboxylated ones. Surprisingly, alkylated rhamnolipids were more favourably inserted into model membrane suggesting that the differences in the biological activity could be derived from a stronger interaction of alkylated rhamnolipids with the domain boundary regions within the membrane where the elicitor is inserted^69^. It is known that the effect of fengycin, orfamide or surfactin lipopeptides on the cell plasma membrane varies from transient permeabilization to solubilisation^70–72^. We observed that SRBs induced changes in plant plasma membrane permeability depending on the concentration and the chain length. This process is unlikely to lead to strong membrane damages, as we did not observe phytotoxic effects on the plants even at high concentration over a long period of time (several days). Interestingly, the laminarin sulfate elicitor PS3 also induced electrolyte leakage in tobacco leaves without inducing cell death and was proposed to interact with plasma membranes^73^. Yeast elicitors consisting in a mixture of glucan, mannan, and chitin, were shown to exhibit pore-forming properties resulting in ion fluxes^74^. This pore-forming activity could explain the electrolyte leakage caused by SRBs.

In the present work, we show that the synthetic elicitor SRB_14i_ is able to induce early immune responses, defence gene activation and enhanced plant protection against *Pst* that dependent on the SA pathway. This fact opens the door to a wide array of new synthetic elicitors by generating amphiphilic compounds that could participate in broadening the variety of crop protection products available in the agriculture market but also to better understand the molecular mechanisms involved in plant immunity. Moreover, when compared to the difficulty and high cost that purification of microbial elicitors involves, chemical synthesis proved to be easy to scale-up at industrial level with affordable costs and provides the additional advantage of making possible structural modification, in order to adapt the final product to new uses.

## Materials and Methods

### Synthesis of SRBs

#### Glycosidation of L-rhamnose

To a solution of L-rhamnose (4.0 g, 22.0 mmol) and unsaturated alcohol (2 eq, 44 mmol) were added at 80 °C, 2.5 g of PTSA (0.6 eq, 13.2 mmol) in three portions. After 5 h of reaction, the mixture was neutralized with the addition of a 500 mM sodium methoxide (MeONa) solution (ca. 26 mL) and the purification of the major α anomer (ratio α/β: 95/5) was performed using flash chromatography (eluting mixture: CH_2_Cl_2_/MeOH, 9:1). Yields = 75–85%.

#### General procedure for the preparation of the rhamnoside based bolaamphiphiles by metathesis

The rhamnoside (10 mmol, 1 eq) was diluted in CH_2_Cl_2_ (40 mL) in a Schlenk tube under argon and the Grubbs’ I catalyst (823 mg, 1 mmol, 0.1 eq) was added in three portions over 3 h. After 8 h of reaction at 45 °C, the solvent is evaporated under reduced pressure and the residue is purified using flash chromatography (eluting mixture: CH_2_Cl_2_/MeOH 9:1). Bolaamphiphiles were obtained with good yields (65–75%).

#### General procedure for palladium catalyzed hydrogenation

SRBs (10 mmol) were dissolved in 25 mL of ethanol under Ar atmosphere. After 10 min, 80 mg of palladium on activated charcoal (Pd/C, 10% w/w) were added and the solution was stirred another 10 min under Ar atmosphere before being submitted to H_2_ flow until completion (24 hours at room temperature). Once the reaction was completed, the reaction mixture was filtered through Celite. The obtained solution was then evaporated under reduced pressure. Saturated SRBs were obtained with a quantitative yield.

#### Plant material and elicitation

*Arabidopsis thaliana* ecotype Col-0 was used as WT parent for all experiments. Seeds from *rbohD, bak1–5-bkk1-1*, *bik1-pbl1* were provided by C. Zipfel. *dorn1-1* seeds were obtained from NASC stock (SALK_042209). All mutants, including *sid2* and *jar1*^9^ are in the Col-0 background. *Arabidopsis* were grown in soil (Gramoflor, Germany) at 21 °C with 60% relative humidity and a 12 h light/12 h dark cycle (light intensity 150 µM/m^2^.s). For RT-qPCR analysis, *Arabidopsis* seedlings were germinated in solid Murashige an Skoog (MS) basal medium with vitamins (pH 5.7) and transferred to 12-well sterile plate containing 1 mL of liquid MS (5 seedling per well) 4 days later. When seedlings were 10-days-old, medium was changed with 1 mL of fresh medium prior elicitation. Purified SRBs were dissolved in 100% ethanol and were used at the concentrations mentioned in the text (from 10 to 300 µM). Final concentration of ethanol for the experiments did not exceed 0.5%, a concentration that was not stressful under our experimental conditions. Control plants were treated with the corresponding final ethanol concentration of the SRB solutions.

For infiltration assays, four weeks-old *Arabidopsis* were syringe infiltrated with corresponding elicitor concentration or ethanol for control. Photographs were taken with a canon Powershot G12 camera 48 h after infiltration.

### Protection assays

For the protection tests against *B. cinerea* (strain B05.10)^9^, 6-weeks-old plants were leaf spray-elicited with 350 µM SRB solution. Two days after elicitation, 7 mm leaf discs were cut and placed in Petri dishes onto water-wet Whatman filter paper. *B. cinerea* was initiated from a silica gel crystal stock and cultivated in PDA plates at 22 °C for 2 weeks. Conidia were collected by adding 4 mL of conidia suspension (KH_2_PO_4_ 1.75 g/L, MgSO_4_ 0.75 g/L, Glc 4 g/L, peptone 4 g/L, Tween 20 0.02% [v/v]) and filtered with cheesecloth to separate hyphae from conidia. The conidia suspension was adjusted to 10^5^ conidia/mL, incubated for 9 h at 22 °C for germination and *Arabidopsis* leaf discs were inoculated with one drop of the conidia suspension. Petri dishes containing the inoculated leaf discs were parafilm-sealed and incubated at 22 °C for 4 days before quantifying the necrotic area with the ImageJ software^75^.

For protection assays against *Pseudomonas syringae* pv. *tomato* DC3000 (*Pst*)^76^, 15 *Arabidopsis* seeds were sawn in order to form a clump. When the clump was 3 to 5-weeks-old, plants were spray-elicited 2 days before pathogen inoculation. *Pst* was cultured overnight at 28 °C in liquid King’s B medium, supplemented with rifampicin (50 µg/mL) and kanamycin (50 µg/mL). Subsequently, bacterial cells were collected by centrifugation at 3000 g for 5 min and resuspended in 10 mM MgCl_2_-Silwet L-77 0.025% to a final optical density of 0.01 (OD_600nm_). For plant inoculation, 3 mL of the bacterial solutions were sprayed on plant leaves (*Arabidopsis* clumps). Mock plants were treated with the same solution free of bacteria. Inoculated plants were incubated in sealed boxes under the same conditions of *Arabidopsis* culture. Three days after inoculation, clump leaves were harvested, grinded in 10 mL of 10 mM MgCl_2_ solution and different dilutions were used to determine the CFU/mg fresh weight on solid KB medium dishes supplemented with antibiotics.

### Bacteriostatic and bactericide assay

After growth in King’s B medium, *Pst* cells were collected by centrifugation at 3000 g for 5 min and resuspended to a final optical density of 0.01 (OD_600nm_). Then corresponding elicitor or ethanol was added to this solution, which was subsequently distributed in 96 wells plate. OD_600nm_ was monitored every hour during 48 h with a TECAN F200 pro. The bactericide effect of elicitor was monitored by counting CFU 24 h post-treatment. To this end, King’s B medium containing *Pst* supplemented with elicitor or ethanol was serial diluted in 10 mM MgCl_2_ and CFU/mL was counted on solid KB medium dishes supplemented with antibiotics.

### Extracellular ROS production

Five mm leaf disks or petioles of 6-weeks-old *Arabidopsis* plants were incubated overnight in 96-wells plates containing 150 µL of distilled water at room temperature. Samples were then rinsed with distilled water and challenged in darkness with a luminol-horseradish peroxidase solution^65^ containing SRBs at the concentrations mentioned in the figure’s legends. ROS production was monitored with a Tecan Infinite F200 PRO (Tecan) every 2 min. For each time point, measurement is integrated over a 2 s period and results were expressed in Relative Light Units (RLUs).

### MAPK phosphorylation assays

Leaf disks (9 mm diameter) from 4 to 6-weeks-old *Arabidopsis* plants were incubated in distilled water for 8 h. SRBs were added to the medium and shock-frozen in liquid nitrogen 15, 60, or 180 min post-elicitation. Following, leaf disks were grinded in a homogenizer Potter-Elvehjem with extraction buffer (0.35 M Tris-HCl pH 6.8, 30% (v/v) glycerol, 10% (v/v) SDS, 0.6 M DTT, 0.012% (w/v) bromophenol blue) (w/v), boiled for 7 min at 95 °C, centrifuged at 11 000 g for 5 min and 30 μL of the supernatant were loaded on SDS-PAGE 12% gel for migration. Then, proteins were transferred to a PVDF membrane with iBLOT gel transfer system (ThermoFisher Scientific) for 10 min at 25 V. For western blot analysis, membranes were blocked for 30 min with 5% low-fat dry milk in TBS-Tween-20 (137 mM NaCl, 2,7 mM KCl, 25 mM Tris-HCl), Tween-20 0.05% (v/v)) and incubated overnight at 4 °C with rabbit polyclonal primary antibodies against phospho-p44/42 MAPK (Cell Signaling, 1:2000). Membranes were washed 3 times with 3% low-fat dry milk in TBS-Tween-20 and incubated for 1 h with anti-rabbit IgG HRP-conjugated secondary antibodies (Bio-Rad, 1:3000) at room temperature. Finally, washed membranes were revealed with SuperSignal^®^ West Femto using odyssey (ODYSSEY^®^ Fc Dual-Mode Imaging System, LI-COR). To normalized proteins, membranes were stripped 15 min with 0.25 M NaOH, blocked with 5% low-fat dry milk in TBS-Tween-20 and immunoblotted with plant monoclonal anti-actin primary antibodies (CusAb, 1:1000) and anti-mouse IgG HRP-conjugated secondary antibodies (Cell Signaling, 1:3000) and revealed as previously described.

### RT-qPCR gene analysis

Plants were harvested at 0 and 9 h post-elicitation, frozen in liquid nitrogen and stored at −80 °C until RNA extraction. RNA extraction and Real-Time qRT-PCR were performed as described previously^9^. For each experiment, PCR was performed in duplicate, and at least two independent experiments were analyzed. Transcript levels were normalized using *AtTubulin* and *AtUbiquitin5* genes as internal controls. Fold induction compared with 0 h post-treatment sample was calculated using the ΔΔCt method. The gene-specific primers used in the present work were *AtUbiquitin5* (F,5′-GGAAGAAGAAGACTTACACC; R,5′-AGTCCACACTTACCACAGTA), *AtTubulin* (F,5′-TGTTCAGGCGAGTGAGTGAG; R,5′-ATGTTGCTCTCCGCTTCTGT), *AtCYP71A12* (F,5-CGAAAGCGAGAAGAGTATTGGA; R,5′-TGTGGCCTAATGGTTGACCG), *AtPDF1.2* (F,5′-CGCACCGGCAATGGTGGAAG; R,5′-CACACGATTTAGCACCAAAG), *AtNPR1* (F,5′TCTTGCCGATGTCAACCATA; R, 5′-CGATCATGAGTGCGGTTCTA).

### Electrolyte leakage

The assay was performed as previously described^65^ with slight modification. Eight leaf discs of 6-mm-diameter were incubated in distilled water overnight. One disc was transferred into 1.5 mL tube containing fresh distilled water and the corresponding elicitor concentration or ethanol for the control. Conductivity measurements (three to four replicates for each treatment) were then conducted over time using a B-771 LaquaTwin (Horiba) conductivity meter.

### Isothermal titration calorimetry (ITC)

Isothermal titration calorimetry measurements were performed on a VP-ITC Microcalorimeter (Microcal). SRB_14i_ titrations were carried out by injecting 10 µL aliquots of large unilamellar vesicles (LUVs) made with PLPC/sitosterol (80/20–5 mM) into the calorimeter cell (V_cell_ = 1.4565 mL) containing SRB_14i_ at 100 µM at constant time intervals of 5 min and at 26 °C. The solution in the sample cell was stirred at a speed of 305 rpm. The reference cell was filled with milliQ water. Prior each analysis, all solutions were degassed using sonicator bath. LUVs dispersion and SRB_14i_ solution were prepared in Tris 10 mM buffer at pH 7.4. Initially, SRB_14i_ stock solution (100 mM) was prepared in DMSO. A 1000× dilution of the SRB_14i_ stock solution was performed in the buffer. An appropriate amount of DMSO was added in the LUV dispersion in order to avoid artifact due to the presence of DMSO.

### Adsorption into a lipid monolayer

An automated LB system (KSV minitrough, KSV instruments Ltd. 75 × 160mm^2^ – volume of 80 cm³) equipped with a home-made injection setup was used as described previously^77^. During the entire duration of the experiment, the subphase was stirred using two cylindrical micromagnetic rods (8 × 1.5 mm^2^) and two electronic stirrer heads located beneath the trough (model 300, Rank Brothers). An autoreversing mode with slow acceleration and a stirring speed of 100 rpm was selected. PLPC or sitosterol monolayers were prepared at the interface of the Langmuir trough filled with 10 mM Tris buffer at pH 7.4 and 22 ± 1 °C. The defined initial surface pressure of these monolayers was obtained by spreading a precise volume of PLPC or sitosterol solutions prepared in chloroform/methanol (2:1 v/v). As soon as the initial surface pressure was stabilized (∼20 min), SRB_14i_ solubilized in DMSO was injected into the subphase to a final concentration of 4.5 µM. After the injection of SRB_14i_, the increase in surface pressure was recorded. Pure DMSO injections into the subphase did not modify the initial surface pressure of the lipid monolayers.

### Permeability assays on liposomes

Membrane permeabilization was followed as described previously^78^. Release of entrapped calcein at self-quenching concentrations from LUV composed by PLPC/sitosterol (80/20–16 µM) can be monitored by the fluorescence increase upon dilution following their leakage from the vesicles. LUVs dispersion was prepared in Tris 10 mM buffer at pH 7.4 as previously described^79^.

SRB_14i_ was added from a stock solution in DMSO and fluorescence intensities were immediately recorded. The percentage of calcein released was defined as [(Ft- Fcontr)/(Ftot- Fcontr)]/100, where Ft is the fluorescence signal measured after 15 min in the presence of SRB_14i_, Fcontr is the fluorescence signal measured at the same time for control liposomes, and Ftot is the total fluorescence signal obtained after complete disruption of the liposomes by 0.1% Triton X-100. All fluorescence determinations were performed at room temperature on a Perkin Elmer LS-50B Fluorescence Spectrophotometer (Perkin-Elmer) using λ_exc_ of 450 nm and a λ_em_ of 512 nm.

### Data Availability

The datasets generated during and/or analysed during the current study are available from the corresponding author on reasonable request.

## Electronic supplementary material

supplementary information
