# Supplementary material for: Synthetic Rhamnolipid Bolaforms trigger an innate immune response in Arabidopsis thaliana
Source: Sci Rep. 2018 Jun 4;8:8534. doi: 10.1038/s41598-018-26838-y (PMC5986815; doi:10.1038/s41598-018-26838-y)
Supplement: Supplementary file 1 — supplementary information [file 41598_2018_26838_MOESM1_ESM.pdf]

Synthetic Rhamnolipid Bolaforms trigger an innate immune response in *Arabidopsis thaliana*

W. Patricio Luzuriaga-Loaiza[1,3,4+], Romain Schellenberger[1+], Yannick De Gaetano[2,5], Firmin Obounou Akong[2], Sandra Villaume[1], Jérôme Crouzet[1], Arnaud Haudrechy[2], Fabienne Baillieul[1], Christophe Clément[1], Laurence Lins[3], Florent Allais[5], Marc Ongena[4], Sandrine Bouquillon[2], Magali Deleu[3] and Stephan Dorey[1*]

Supplementary information

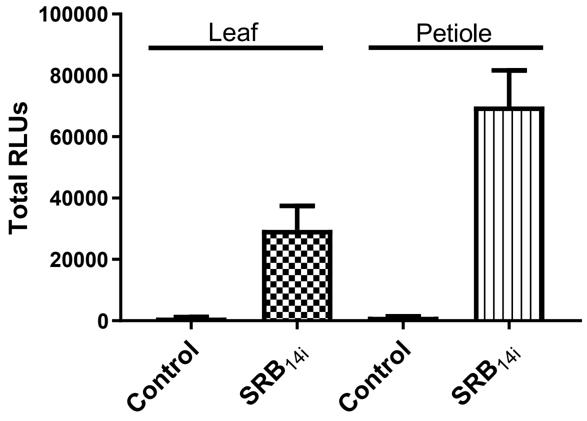

Supplementary figure S1: Extracellular ROS production of *Arabidopsis* leaves or petioles upon elicitation with SRBs. Leaf petioles or leaf disks of 6-weeks-old wild type *Arabidopsis* plants were placed in a 96-wells plate and incubated in water overnight prior SRB_{14i} elicitation. For ROS monitoring, a luminol-peroxidase solution containing 350 µM of SRB_{14i} was added to each well. The luminescence was read immediately after elicitation every 2 min with a Tecan Infinity F200 PRO for 720 min. Histograms were calculated as the total RLUs over 12 hours of monitoring. Data presented are means of at least triplicate experiments ± standard error of the mean (SEM) with n = 6 for each experiment.

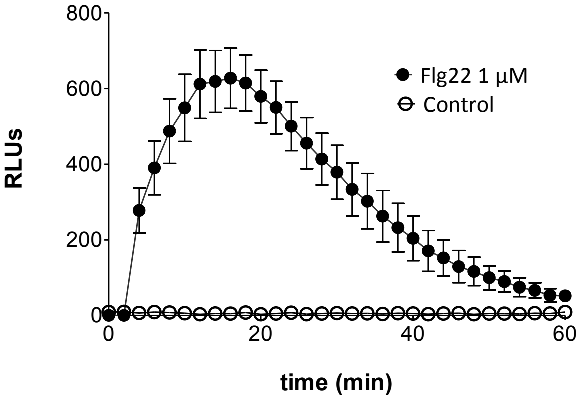

Supplementary figure S2: ROS production induced by 1 µM flg22 or 0.5% ethanol (control). Data presented are means and SEM of at least triplicate experiments with n = 6 for each experiment.

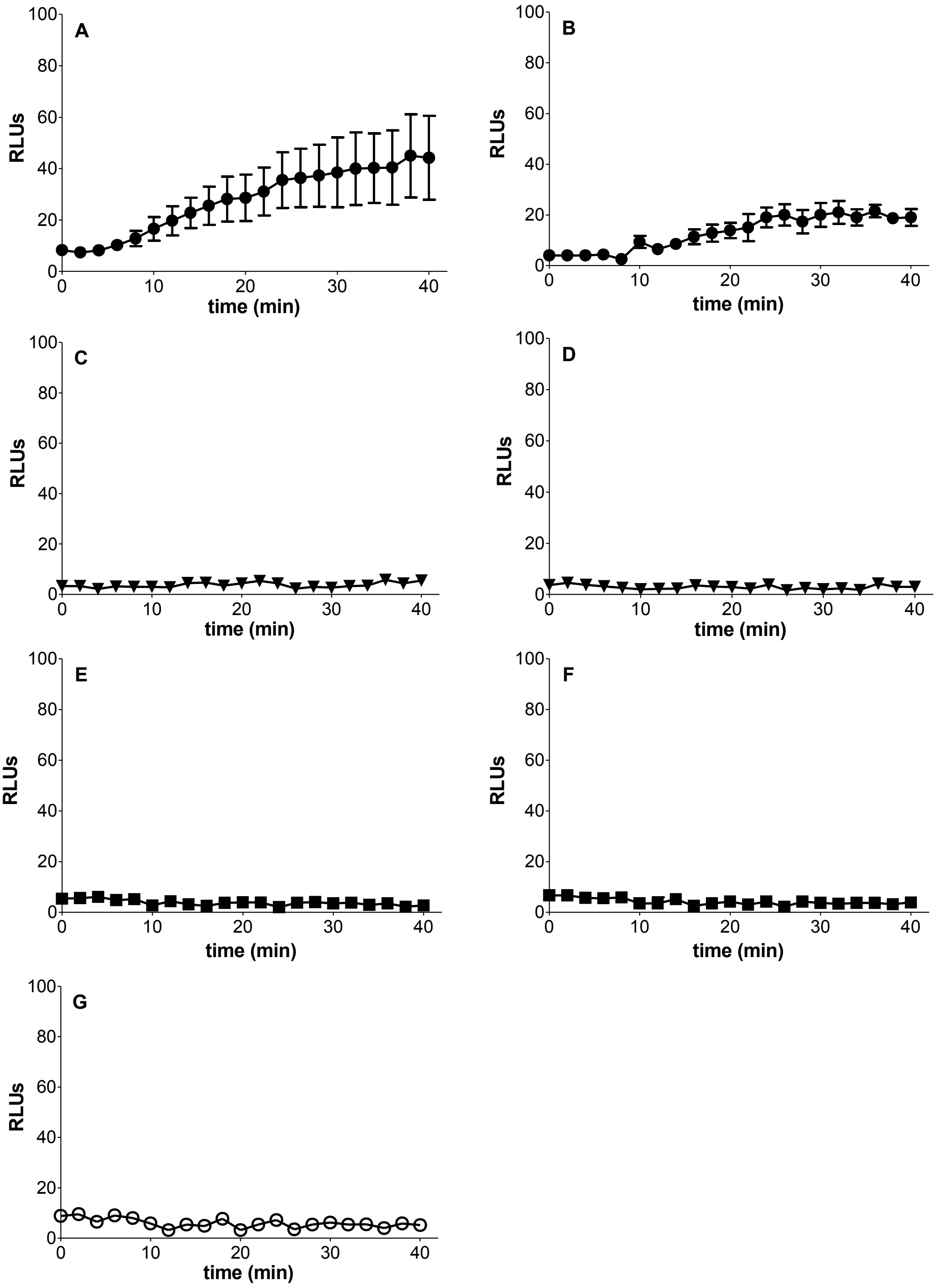

Supplementary figure S3: Extracellular ROS production upon elicitation of *Arabidopsis* with SRBs. Leaf petioles of 6-weeks-old *Arabidopsis* plants were placed in a 96-wells plate and incubated in water overnight prior SRB elicitation. For ROS monitoring, a luminol-peroxidase solution containing 350 µM of the corresponding SRB or solvent (0.5% ethanol) was added to each well. All SRBs contain the same amount of ethanol. The luminescence was read immediately after elicitation every 2 min with a Tecan Infinity F200 PRO for 40 min. Data presented are means of at least triplicate experiments and SEM with n = 6 for each experiment. A) C) E) unsaturated SRB_{14i}, SRB_{10i} and SRB_{18i}, respectively. B) D) F) saturated SRB_{14}, SRB_{10} and SRB_{18}, respectively. G) Control (ethanol).

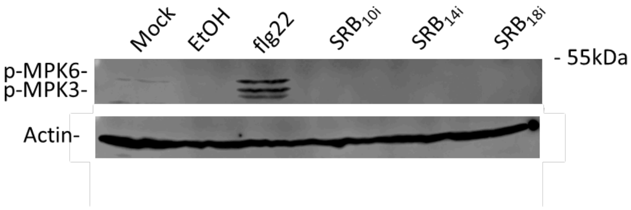

Supplementary figure S4: MAPK3 and 6 phosphorylation of SRBs-elicited *Arabidopsis*. Leaf discs of *Arabidopsis* were elicited with 100 µM of unsaturated SRBs, 1µM of flg22, 0.5% ethanol or water (mock) for 15 min. Kinase activation is shown by immunoblot analysis using an anti-p44/42-ERK antibody. Individual MPKs are identified by molecular mass and indicated by arrows. Anti-actin antibodies were used for protein quantification for each sample. Experience has been done twice with similar results.

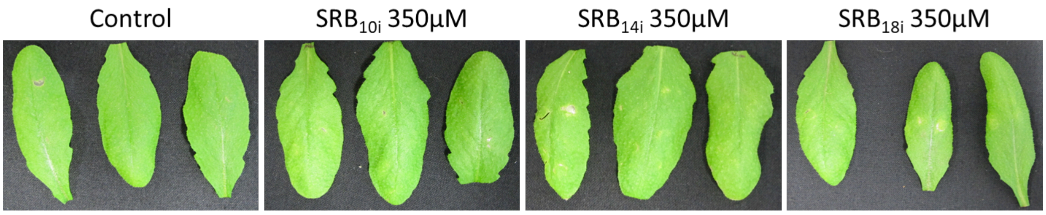

Supplementary figure S5: Effect of SRBs infiltration in Arabidopsis leaves. 350µM of SRBs or control (EtOH 0.5%) was infiltrated in *Arabidopsis* leaves. Photography was taken 48h post infiltration. Experiments have been repeated two times with similar results.

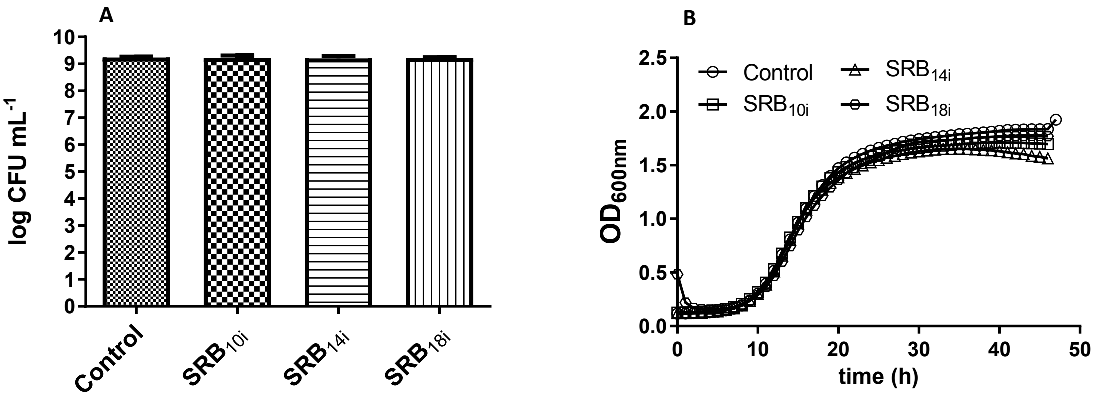

Supplementary figure S6: Direct effect of SRB_i on the growth of *Pst* DC3000 at 350 µM or 0.5% ethanol (Control). *Pst* DC3000 were growth on King's B liquid medium containing the corresponding concentration of SRBs or ethanol. A) The medium was serially diluted 24 hours post treatment and plated on solid King's B for CFU counting. Results represent the mean and SEM of one representative experiment with $n \geq 5$. The differences were analyzed by the non-parametric Kruskal-Wallis test ($p<0.05$) followed by Dunn's multiple comparison test. B) OD_{600nm} was read immediately after addition of molecules every hour with a Tecan Infinity F200 PRO for 48 h. Data presented are from one representative experiment with $n = 6$. Experiments have been realized at least two times with similar results.

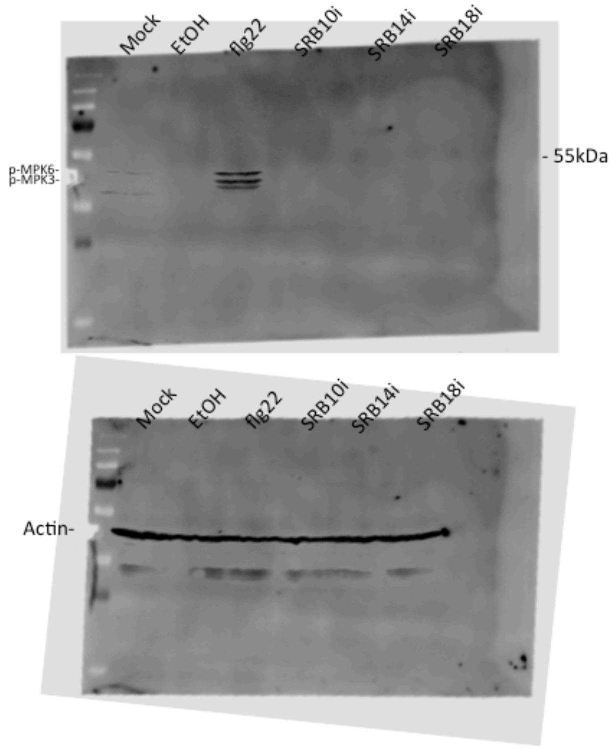

Supplementary figure S7: Originals full-length blots of figure S4.

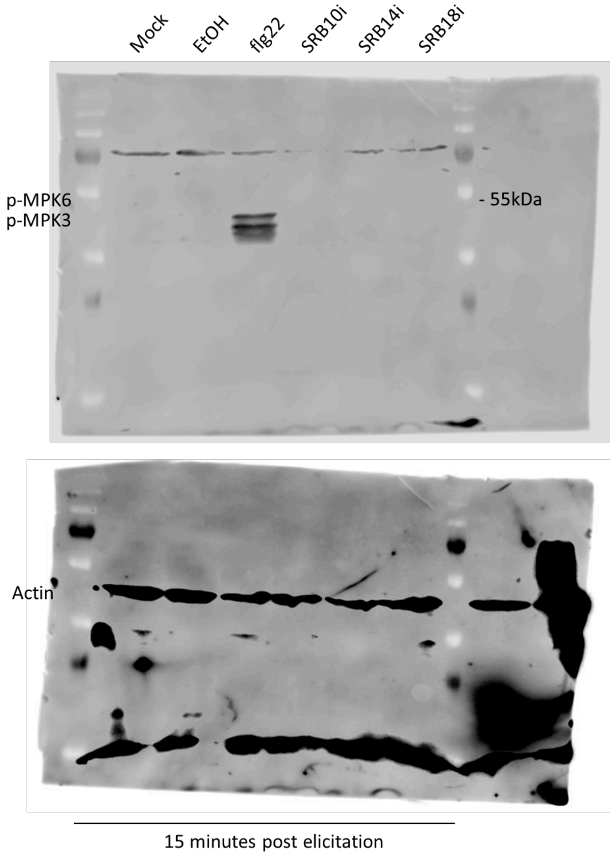

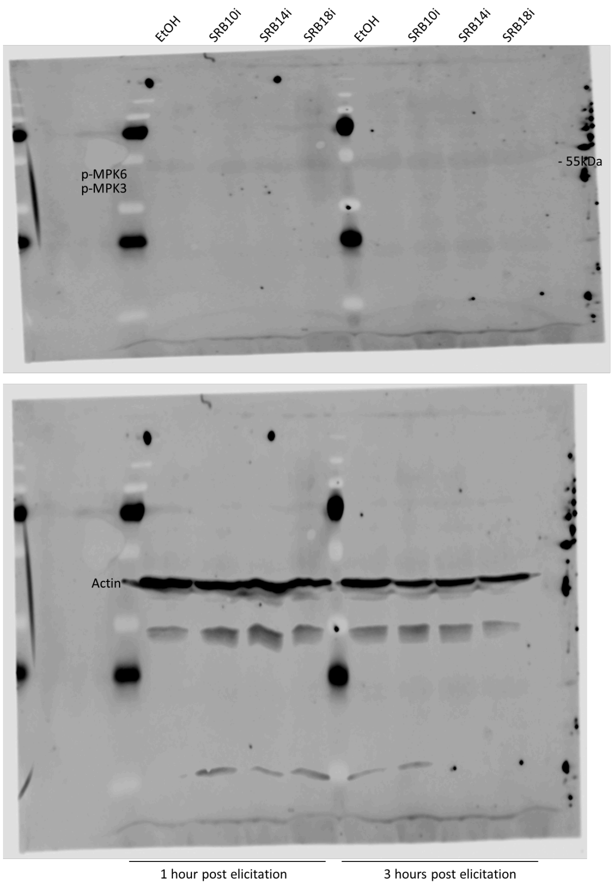

Supplementary figure S8: Originals full-length blots of figure 6.
